# Hyperactivation of DNA-PK by Double-Strand Break Mimicking Molecules Disorganizes DNA Damage Response

**DOI:** 10.1371/journal.pone.0006298

**Published:** 2009-07-21

**Authors:** Maria Quanz, Danielle Chassoux, Nathalie Berthault, Céline Agrario, Jian-Sheng Sun, Marie Dutreix

**Affiliations:** 1 Institut Curie, Hôpital, Département de transfert, Orsay, France; 2 CNRS, UMR2027, Orsay, France; 3 DNA Therapeutics, Evry, France; 4 Muséum National d'Histoire Naturelle, USM503, Paris, France; 5 INSERM, U565, Paris, France; 6 CNRS, UMR 5153, Paris, France; National Institute on Aging, United States of America

## Abstract

Cellular response to DNA damage involves the coordinated activation of cell cycle checkpoints and DNA repair. The early steps of DNA damage recognition and signaling in mammalian cells are not yet fully understood. To investigate the regulation of the DNA damage response (DDR), we designed short and stabilized double stranded DNA molecules (Dbait) mimicking double-strand breaks. We compared the response induced by these molecules to the response induced by ionizing radiation. We show that stable 32-bp long Dbait, induce pan-nuclear phosphorylation of DDR components such as H2AX, Rpa32, Chk1, Chk2, Nbs1 and p53 in various cell lines. However, individual cell analyses reveal that differences exist in the cellular responses to Dbait compared to irradiation. Responses to Dbait: (i) are dependent only on DNA-PK kinase activity and not on ATM, (ii) result in a phosphorylation signal lasting several days and (iii) are distributed in the treated population in an “all-or-none” pattern, in a Dbait-concentration threshold dependant manner. Moreover, despite extensive phosphorylation of the DNA-PK downstream targets, Dbait treated cells continue to proliferate without showing cell cycle delay or apoptosis. Dbait treatment prior to irradiation impaired foci formation of Nbs1, 53BP1 and Rad51 at DNA damage sites and inhibited non-homologous end joining as well as homologous recombination. Together, our results suggest that the hyperactivation of DNA-PK is insufficient for complete execution of the DDR but induces a “false” DNA damage signaling that disorganizes the DNA repair system.

## Introduction

Ionizing radiation (IR) randomly causes damage to all cellular components and induces a large variety of DNA lesions [1], [2]. To ensure efficient repair, eukaryotic cells activate a signaling network that coordinates the rapid detection of DNA damage, cell cycle delay and DNA repair. Correlation between the specific DNA lesions produced by ionizing radiation and these biological endpoints has not been well established. A primary event in this DNA damage response (DDR) is the rapid phosphorylation of histone H2AX (γ-H2AX) in the chromatin micro-environment surrounding a double strand break (DSB) by the phosphatidylinositol 3-kinase protein kinase-like (PIKK) family members ATM, ATR or DNA-PK [3], [4]. Many components of the DDR such as the Mre11/Rad50/Nbs1 (MRN) complex, 53BP1, Brca1, MDC1, ATM and Rad51 form microscopically discernible foci that co-localize with γ-H2AX [5]-[9]. Although γ-H2AX is not essential for the initial recognition of damage by signaling proteins, it seems to be indispensable for their sustained sequestration in the vicinity of DNA lesions [10].

The DDR response can be envisioned as a signal transduction cascade in which DNA lesions act as initial signals that are detected by sensors and passed through transducers [11], [12]. The PIKK kinases have been shown to play prominent roles in the early stage of the DDR by phosphorylating a large set of proteins including chromatin structural proteins, proteins that function in chromosomal repair and maintenance, proteins of the cell cycle checkpoints and some transcription factors. Phosphorylation of the DDR effectors leads to cell cycle arrest, enhanced DNA damage repair and eventually to apoptosis. ATM, ATR and DNA-PK may signal different although partially overlapping types of DNA damage and they share many common effectors. Moreover, they can interact with each other directly or indirectly and thus regulate the each other's activities [13]-[15]. This complexity renders the general picture of the DDR cascade relatively elusive.

Here, we used short and stabilized DNA molecules (Dbait) that mimic DSB to address the specific role of DSB signaling in DDR. In a previous study [16], we used Dbait molecules to sensitize xenografted tumors to radiotherapy. Our results suggested that they are recognized as DNA damage and disorganize DNA repair. We show here that these molecules provide a unique tool to inducing a DSB-specific response in a cell without perturbing replication or introducing other types of damage.

## Results

### DNA-PK activation by Dbait molecules

**Figure 1:**
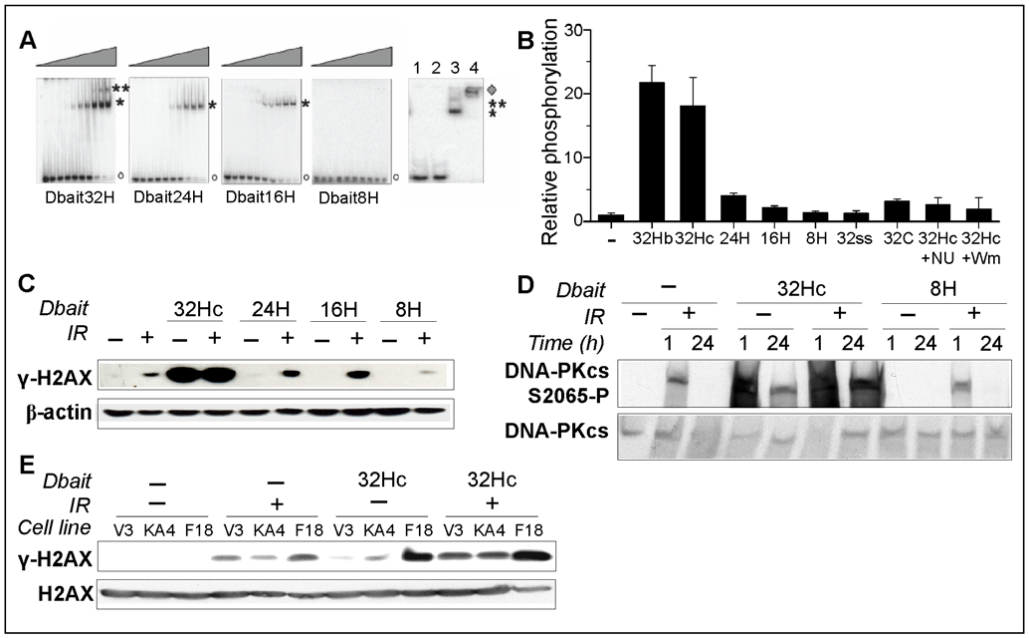
DNA-PK activation and H2AX phosphorylation by Dbait treatment. (A) Various ^32^P-labeled Dbait molecules (32H, 24H, 16H, 8H), were incubated with increasing amounts (0-320 ng/ml) of Hep-2 nuclear extract and analyzed on polyacrylamide gels. Asterisks indicate one or two dimer shift. Antibodies against Ku80 were added prior to migration to confirm by supershift that the bands indicated by asterisks contained Ku80 protein: Lane 1, 32H; lane 2, 32H + anti-Ku80; lane 3, 32H + nuclear extract; lane 4, 32H + nuclear extract + anti-Ku80. The diamond indicates the supershifted band of Dbait–Ku complexes bound by anti-Ku80. (B) Stimulation of DNA-PK kinase activity by 20 mM of various Dbait molecules was measured in 1.5 µg HEp-2 nuclear extract. When indicated, inhibitors NU7026 (NU) and wortmannin (Wm) were preincubated with extract 5 min prior to 32H addition (hatched). Data represent the mean value and standard deviation of at least three independent experiments. (C-E) Cells were transfected (5 h) with different Dbait molecules as indicated and irradiated (10 Gy) or not. If not indicated otherwise, proteins were extracted 1 h after end of treatment for western blot analysis. (C) HEp-2 cell lysates were probed for γ-H2AX and β-actin. (D) Immunoblot of phosphorylated DNA-PKcs; total DNA-PKcs was revealed on a separate membrane. (E) V3 (DNA-PKcs^-/-^), KA4 (DNA-PKcs kinase deficient) and F18 cells (DNA-PKcs proficient) were treated as indicated and the lysates were probed for γ-H2AX and total H2AX.

We first screened for the smallest Dbait molecules that could be detected as DSBs in a cell. Since binding of Ku proteins followed by DNA-PKcs recruitment and activation of its kinase activity are the earliest events in DSBs repair by NHEJ, we analyzed the minimal requirements to trigger these steps using various short DNA molecules mimicking DSBs (Dbait). The Dbait molecules used were hairpin double-stranded DNA with one blunt end and various sequences or lengths (listed in supplementary Fig. S1). To improve the stability and persistence of these molecules within the cell, the two complementary strands were linked by a hexaethylene glycol linker (H) at one end and protected from exonuclease attack by substituting the three 3′ and 5′ terminal nucleotide residues with phosphorothioate nucleotides at the other end [17]. The formation of the DNA-PK complex (Ku70/Ku80/DNA-PKcs) with the various Dbaits in cell extracts was monitored by gel shift assays (Fig. 1A) and DNA-PK kinase activity (Fig. 1B). Binding of Ku proteins revealed a migration shift corresponding to one heterodimer for 16-bp (16H) or 24-bp (24H) Dbait molecules and two heterodimers for the 32-bp (32H) Dbait. Only the 32-bp molecules, irrespective of their sequence were (32Hb, 32Hc) were able to induce DNA-PK kinase activity (Fig. 1B). The 16H and 24H molecules that bound only one Ku heterodimer were almost as inefficient as the short 8-bp (8H) Dbait that did not bind Ku. Note that neither a 32 bases long single strand molecule (32ss) nor a ‘dumbbell’ 32-bp dsDNA fragment (32C), in which both ends were tethered by two hexaethylene glycol loops, activated kinase activity of purified DNA-PK (data not shown) or extract (Fig. 1B). The Dbait-mediated DNA-PK activation was abolished upon the addition of wortmannin (Wm), an inhibitor of the phosphoinositide 3-kinases, or the addition of NU7026 (NU), a specific inhibitor of DNA-PK [18]. *In vitro*, DNA-PKcs is rapidly autophosphorylated upon activation. Many *in vitro* and *in vivo* phosphorylation sites of DNA- PKcs have been identified thus far, including the Thr-2609 cluster and Ser-2056 and phosphorylation sites. Ser-2056 cluster activation seems to be specifically dependent upon DNA-PKcs activation [19]. In agreement with kinase activation in crude extract, we found that DNA-PKcs was phosphorylated at Ser2056 in cells treated with 32Hc in MRC5 ( Fig. 1D) as well as in the ATM deficient fibroblasts AT5BI (data not shown).

In higher eukaryotic cells, the appearance of γ-H2AX in cell nuclei is often used as an indicator of the presence of DNA DSBs produced by IR [20]. Therefore, we investigated whether the DSB-mimicking Dbait molecules would lead to H2AX phosphorylation in cells. Transfection of HEp-2 cells followed by immunoblotting of cell extracts confirmed that, in agreement with the above described DNA-PK activity test, a length of 32-bp was required to activate H2AX phosphorylation (Fig. 1C). The γ-H2AX signal induced by 32Hc was six to seven-fold higher than that induced by 10 Gy IR as quantified from western blot. ATM and DNA-PK are primarily responsible for the phosphorylation of H2AX (γ-H2AX) at DSBs. We have shown that the response to 32Hc is independent of ATM as it is not compromised in the ATM-deficient cell line AT5BI [16]. To confirm the central role of DNA-PKcs kinase activity in the cellular response to 32Hc, we tested for the induction of DDR in CHO derived cell lines lacking DNA-PKcs (V3) and complemented with vectors expressing the wild-type protein (V3-F18) or a kinase-dead mutant of DNA-PKcs (V3-KA4). The cell line V3-KA4 (K3752R) expresses mutant DNA-PKcs at levels comparable to the recombinant wild-type V3-F18 cells (Kurimasa et al. 1999). In contrast to irradiation, 32Hc required DNA-PKcs kinase activity to induce efficient H2AX phosphorylation. The V3-KA4 mutant was as defective as V3 cells that do not express DNA-PKcs in phosphorylating γ-H2AX after 32Hc treatment (Fig. 1E). The dependence of the 32Hc response upon DNA-PKcs kinase activity was further confirmed by specific inhibition by wortmannin and NU7026 of γ-H2AX induction in various cell lines (supplementary Fig. S2A). Caffeine, which is more specific of ATM or ATR, had no effect on the response to 32Hc treatment. These data suggest that the phosphorylation induced after 32Hc transfection is essentially promoted by DNA-PK activation. In agreement with 32Hc activity requirement for DNA-PKcs kinase, we found that DNA-PKcs proficient cell lines M059K and F18 were radiosensitized by transfection with 32Hc while DNA-PKcs deficient cell lines M059J, KA4 and V3 were not (Supplementary Fig. S2B).

### 32Hc induces persistent kinase activation in transfected cells

**Figure 2:**
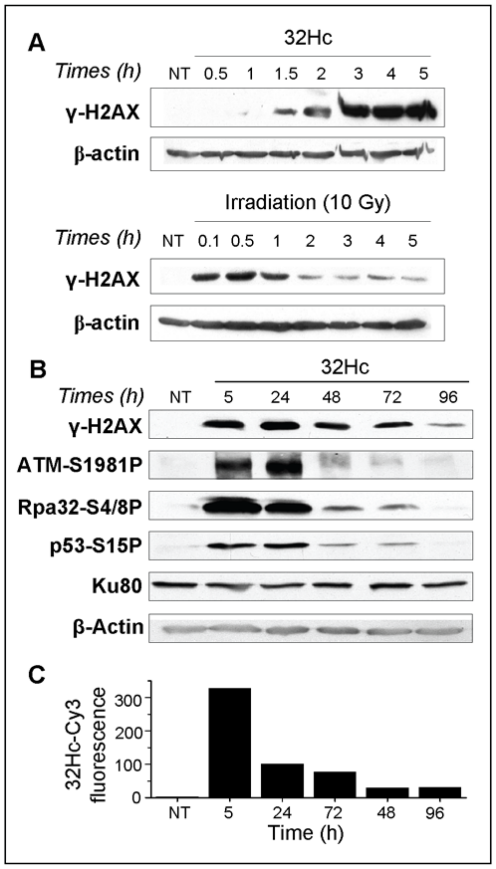
Kinetics of the 32Hc induced response. (A) MRC-5 cells were transfected or irradiated at time 0 and then extracted for western blot analysis of γ-H2AX and β-actin at the indicated times. (B-C) To monitor the kinetics of DNA-PK target protein phosphorylation and 32Hc elimination, cells were transfected with Cy3-conjugated 32Hc and (B) one part of each sample was processed for SDS-PAGE and hybridized with antibodies against β-actin, Ku80, and phosphorylated p53, Rpa32 and H2AX. Phosphorylated ATM was revealed on a separate gel due to its higher molecular weight. (C) In parallel, the other part of the sample was analyzed by flow cytometry for the mean Cy3-fluorescence.

We studied the kinetics of H2AX phosphorylation in cell cultures transfected by 32Hc molecules. The γ-H2AX concentration increased with time to reach a maximum 4-5 h after beginning 32Hc transfection (Fig. 2A, upper panel) and remained at the same level over the following 24 h (Fig. 2A-B), whereas the γ-H2AX signal after IR diminished rapidly within 2 h (Fig. 2A, lower panel). 32Hc-induced γ-H2AX was still detectable four days after transfection (Fig. 2B). The persistent phosphorylation of DDR effectors was not restricted to H2AX but was also observed for other DNA-PK kinase targets such as Rpa32 (S4/8), p53 (S15) and ATM (S1981), which have been shown to be transiently phosphorylated after irradiation (Fig. 2B). Such prolonged phosphorylation has not previously been observed after irradiation or other DNA-damaging treatments [21]. These results indicate that 32Hc promotes a persistent DDR-like kinase activation in transfected cells that slowly decreases with time.

To determine the correlation between cellular 32Hc content and the induced DDR effector-phosphorylation, we monitored the kinetics of the fluorescent Dbait 32Hc-Cy3 cellular content by flow cytometry (Fig. 2C). The mean concentration of 32Hc-Cy3 was maximal 5 h after beginning the transfection and decreased regularly during the days following transfection. The 32Hc-induced phosphorylation did not decay immediately with 32Hc molecules concentration in the cells. The level of γ-H2AX was still maximal 24 h after transfection when the cellular 32Hc-Cy3 content had already decreased to 30% of its initial concentration. However residual 32Hc-Cy3 was still detectable four days after treatment and could contribute to maintaining phosphorylation activity.

### 32Hc-dependent kinase activation leads to an “all-or-none” phosphorylation response

**Figure 3:**
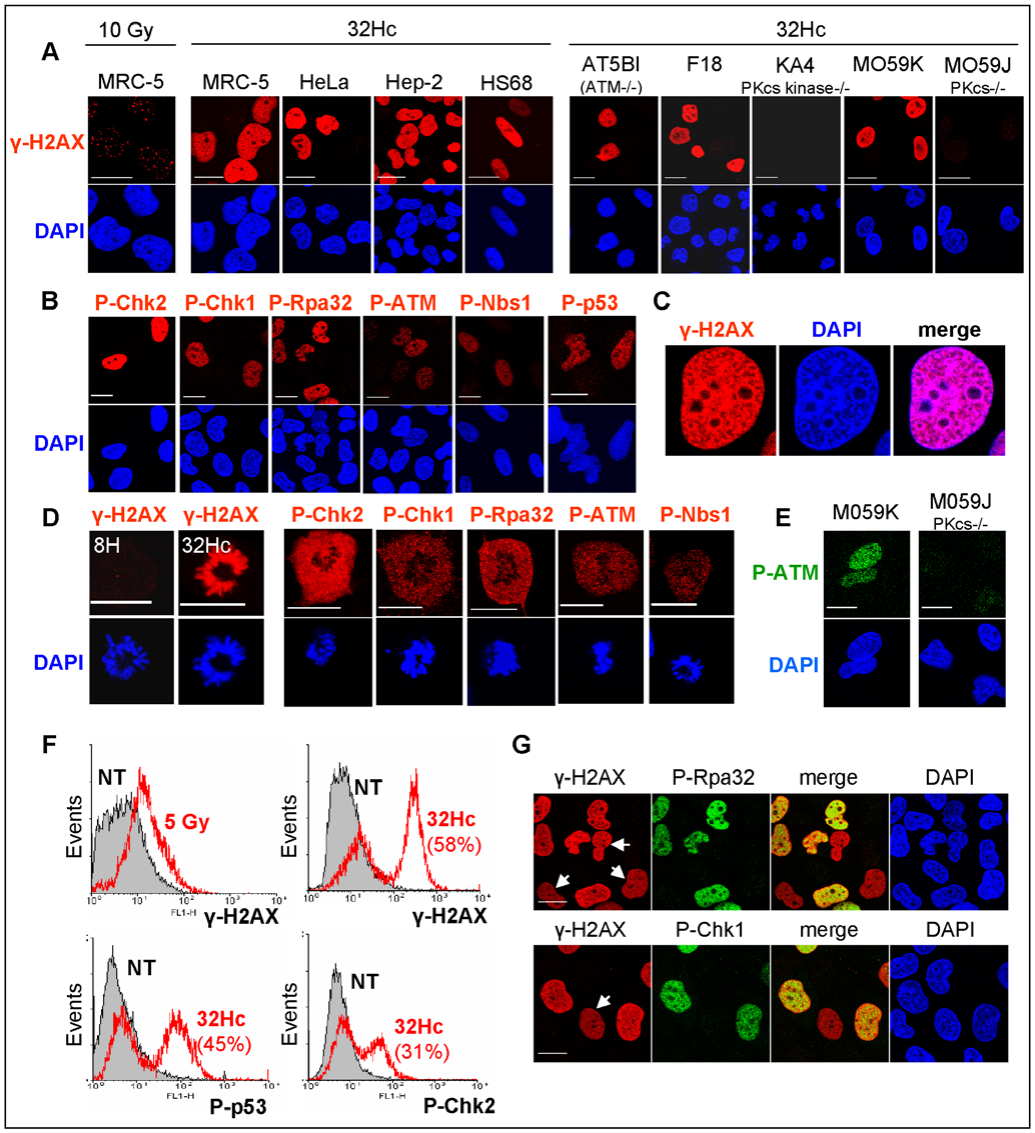
Phosphorylation pattern of DNA damage signaling proteins in 32Hc treated cells. (A) Immunostaining of γ-H2AX in MRC-5 cells after 10 Gy IR (left panel) and in various cell lines after 32Hc treatment. Cells were fixed 2 h after IR and 1 h after the end of transfection. (B-D) Phosphorylation pattern of different DDR proteins in MRC-5 cells after 32Hc treatment. Cells were immunostained with antibodies against γ-H2AX, Chk2-T68P, Chk1-S345P, Rpa32-S4/8P, ATM-S1981P, Nbs1-S343P, p53-S15P as indicated. (B) Interphase cells. (C) Co-localization of γ-H2AX and DNA (D) M-Phase cells. (E) 32Hc treated M059K and M059J cells were probed for ATM-S1981P. (F) Flow cytometry analysis of not-treated (NT, gray), irradiated (5 Gy, upper left panel) or 32Hc transfected MRC-5 cells with immunostaining for the indicated phosphoproteins. Percentages reflect the proportion of the “positive” cells after 32Hc treatment. (G) Co-staining of γ-H2AX and Rpa32-S4/8P (upper panel) or Chk1-S345P (lower panel). Arrows indicate γ-H2AX positive cells that are negative for the other phosphoprotein. Scale bar: 20 µm.

After irradiation, H2AX is phosphorylated in megabase sized regions at the site of each nascent DSB and can be detected as nuclear foci by specific antibodies. Microscopy (Fig. 3A, left panel) and flow cytometry analyses (Fig. 3F) of irradiated cells show that the γ-H2AX foci content distribute according to a normal distribution within the cells in the population. In contrast, cells treated with 32Hc distribute into two distinct populations: one with a basal level of γ-H2AX similar to the untreated population and the other with a high content (20-fold increased) of γ-H2AX (which we call γ-H2AX positive). Microscopic analyses of the γ-H2AX-positive cells revealed a uniform distribution of γ-H2AX throughout the nucleus excluding nucleoli without visible accumulation into distinct regions. Interestingly, the H2AX phosphorylation pattern correlated almost perfectly with the chromatin distribution (Fig. 1C-D). In contrast, the γ-H2AX foci induced by DNA damaging treatments have been shown to preferentially locate in regions of less densely packed euchromatin [22], [23]. We observed pan-nuclear phosphorylation in 40%-80% of the cells in all PIKK kinase proficient cell lines tested: rodent cells (F18) and human primary fibroblasts (HS68), transformed fibroblasts (MRC-5) or tumor cells (HeLa, HEp-2, M059K) (Fig. 3A). As previously observed by western analysis, we found that DNA-PK deficiency (KA4, M059J) but not ATM deficiency (AT5BI) inhibits γ-H2AX positive cell formation in 32Hc treated cells (Fig. 3A). The other DNA-PK targets such as Rpa32 (S4/8), p53 (S15), Chk1 (S345), Chk2 (T68), Nbs1 (S343) and ATM (T1981) showed similar bimodal responses with a pan-nuclear “all-or-none” phosphorylation pattern (Fig. 3B). Note that ATM phosphorylation at Ser1981 in response to 32Hc treatment was abolished in the DNA-PKcs deficient cell line M059J (Fig. 3E). This suggests a DNA-PK dependence of the phosphorylation at this site instead of stimulation of ATM autophosphorylation by 32Hc molecules. Whereas in M-Phase, all γ-H2AX was located along the condensed chromosomes with no cytoplasmic signal, the other phosphorylated proteins remained in the cytoplasm and did not co-locate with the chromatin indicating that 32Hc-activated DNA-PK does not lead to sequestration of the target proteins at a specific locus (Fig. 3D).

Co-staining of phosphorylated targets indicates that hyper-phosphorylation occurs for different targets in a single cell (Fig. 3G). However, quantitative analysis of the number of positive cells for each target revealed that the size of the γ-H2AX positive subpopulation is higher than the size of the positive subpopulations of the other phosphoproteins (Fig. 3F-G). High γ-H2AX content was detected in all cells positive for another phosphorylated DDR effector, however a subset of the γ-H2AX-positive cells did not contain high levels of phosphorylated Rpa32 or Chk1 (Fig. 3G), suggesting that H2AX is more readily phosphorylated by DNA-PK than other DDR effectors that intervene at a later stage in DNA repair. In the following experiments, we used mainly γ-H2AX detection to discriminate cells with 32Hc induced response.

### Activation of DNA-PK requires a threshold concentration of 32Hc

**Figure 4:**
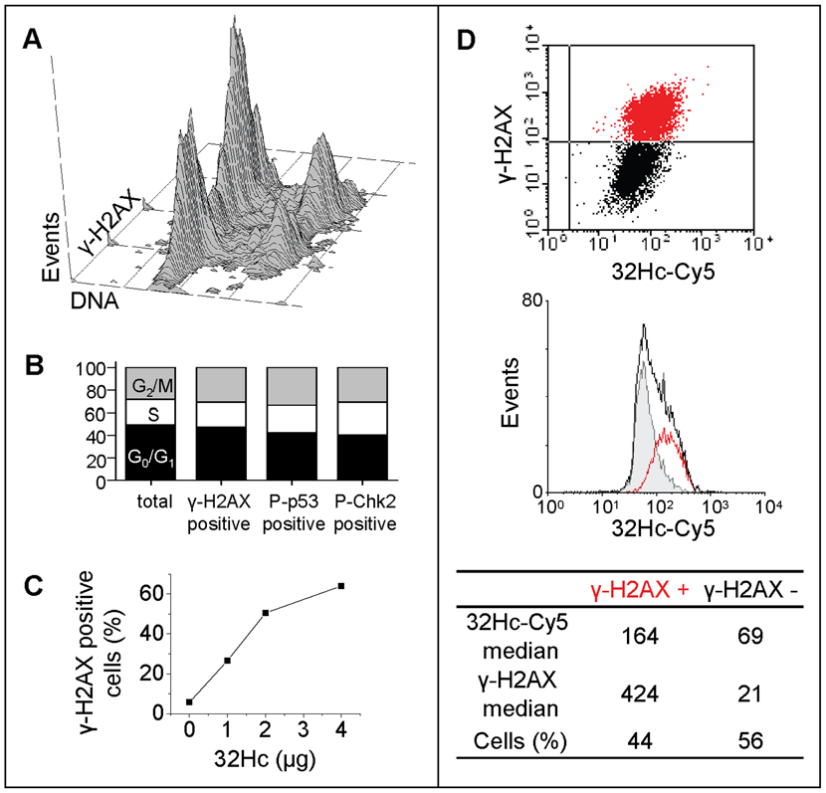
Characteristics of γ-H2AX positive cells. Flow cytometry analysis of 32Hc treated MRC-5 cells co-stained for various phosphorylated proteins and DNA content (A) Distribution of the population stained with γ-H2AX. (B) quantification of the cell cycle distribution of the total cell population and the γ-H2AX, P-p53 or P-Chk2 “positive” subpopulation of the indicated proteins. (C) Percentage of γ-H2AX positive cells in the population (as quantified by flow cytometry) after transfection with varying amounts of 32Hc. (D) Dotplot of cells transfected with Cy5-conjugated 32Hc and immunostained for γ-H2AX (upper panel). Lower panel: 32Hc-Cy5 distribution in the cell population (black) with superposed subpopulations gated for γ-H2AX negative (gray) and positive (red) cells.

The “all-or-none” phosphorylation response observed for all the DNA-PK targets indicates that DNA-PK activation occurs only in a part of the population transfected by 32Hc. We first asked if the induced DDR-like response could be favored in a specific cell cycle phase, corresponding to a subset of the asynchronous exponentially growing population. To test this hypothesis, we performed flow cytometry analysis of MRC-5 cells co-stained with propidium iodide (PI) and anti-γ-H2AX antibody immediately after transfection (Fig. 4A). About 50% of the cells were γ-H2AX positive 1 h after the end of transfection. The positive cells were not significantly enriched in any particular cell cycle phase. The same result was found for p53-S15P and Chk2-T68P positive cells (Fig. 4B).

Another explanation for the differences observed between the different cells in the population could be that the γ-H2AX negative cells did not take up 32Hc. We used the co-detection of γ-H2AX and fluorescent 32Hc to determine the relationship between 32Hc content and the DDR signal. We found that 32Hc molecules distribute uniformly in a cell population following a Gauss law (Fig. 4D) and do not present a bimodal distribution as does the γ-H2AX population (Fig. 3E). However, measurement of 32Hc content in γ-H2AX positive cells indicated that these cells contained in general a higher content of 32Hc than γ-H2AX negative cells in the same population (Fig. 4D). Increasing the amount of 32Hc transfected increased the proportion of γ-H2AX positive cells but did not change the “all-or-none” activation mode (Fig. 4C). Therefore, unlike the dose-response proportionality of the DDR [24], 32Hc induced response seemed to require a threshold amount of DSB signal in order to become activated. This signal “threshold” was not dependent upon the method used for 32Hc transfection since the polyamidoamine dendrimers “Superfect”, the polyethylimine (PEI) linear polymer and electroporation with naked 32Hc molecules resulted in the same pattern of 32Hc-induced H2AX phosphorylation (supplementary material Fig. S3A). Pan-nuclear γ-H2AX patterns have been described previously as a result of massive DSB induction in pre-apoptotic cells [25], [26]. We therefore performed TUNEL assays to test for the presence of DNA strand breaks in γ-H2AX-positive cells (Supplementary Fig. S3B). Since no DNA damage was detectable, we conclude that the hyper-phosphorylation of H2AX is due only to the ectopic activation of DNA-PK by 32Hc molecules and not to DNA fragmentation.

### 32Hc activated response does not lead to cell cycle arrest or apoptosis

**Figure 5:**
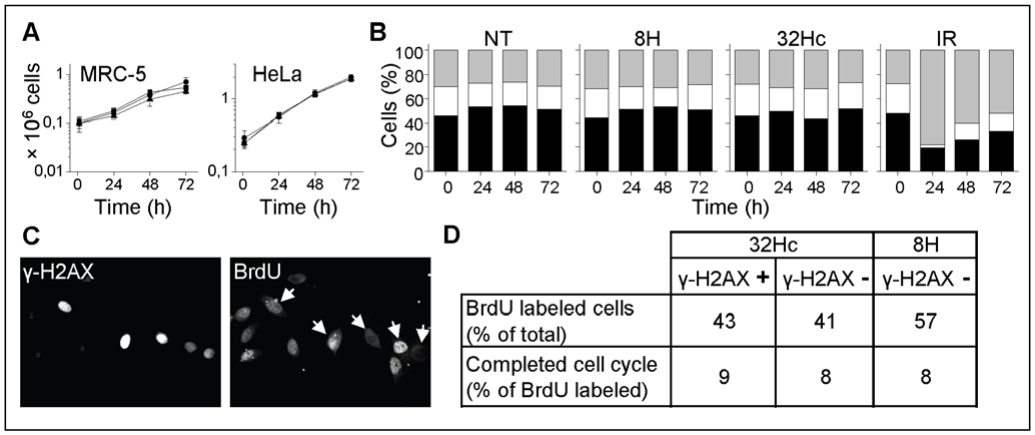
Effect of 32Hc on cell cycle and viability. (A) Proliferation rates of untreated (squares), 8H (circles) or 32Hc (triangles) treated MRC-5 (left panel) and HeLa (right panel) cells. Error bars represent SD. (B) Flow cytometric cell cycle analysis of untreated, 8H or 32Hc transfected and irradiated (10 Gy) MRC-5 cells. DNA was stained with propidium iodide. Cells were rediluted once after 24 h. (C) DNA synthesis was analyzed in HEp-2 cells by pulsing cells with BrdU at 24 h after 32Hc treatment: Left Panel: cells stained with γ-H2AX antibodies; Right Panel: cells stained with BrdU antibodies (arrows indicate γ-H2AX positive cells). (D) The cell cycle rate was estimated by microscopy analysis of HEp-2 cells' nuclei pulsed with BrdU 1h after transfection and grown for 24 h before fixation and γ-H2AX, BrdU co-immunostaining (see material and methods). *In situ* measurement of DNA content and the pattern of BrdU labeling indicated cells having completed a cell cycle.

After irradiation, DDR activation leads to DNA damage repair, cell cycle arrest and apoptosis. Since 32Hc treatment induces a high and persistent phosphorylation of several effectors of the DDR response (such as p53, Chk1, Chk2, ATM), we asked how it could induce cell cycle arrest or apoptosis in phosphorylation-positive cells. We found that cells continued to proliferate at a normal rate for three days following 32Hc transfection (shown exemplarily for MRC-5 and HeLa cells in Fig. 5A). The percentage of γ-H2AX-positive cells in treated populations at 5 h and after one population doubling (24 h) remained the same, indicating that the γ-H2AX-positive cells continue to proliferate (data not shown). Cell cycle analyses by flow cytometry of IP stained MRC-5 (Fig. 5B), HeLa and HEp-2 cells (not shown) transfected with 32Hc revealed neither cell cycle delay nor an increase in the sub-G1 fraction, indicative of apoptotic cells. Furthermore, individual cells were examined microscopically for their BrdU pattern and γ-H2AX staining. Efficient BrdU labeling of γ-H2AX-positive cells when pulsed with BrdU 24 h after 32Hc transfection confirms that these cells distribute in all cell cycle stages and continue replicating (Fig. 5C).

To analyze whether the γ-H2AX-positive cells have the same cell cycle rate as the γ-H2AX-negative cells from the same population or cells transfected with 8H, BrdU labeling was performed at the end of transfection and cells were fixed 24 h after the BrdU pulse. Cells continuing a cycle during incubation after the BrdU pulse may be found at any position of the cycle at the time of fixation. A characteristic feature of the pulse BrdU label is that a given pattern typical of S phase stage (typical of early, mid or late S phase) is retained even if the cell enters a following cell cycle phase [27]-[29]. Comparing BrdU pattern with DNA content indicates if the cell has pursued its cycle and how far: for instance, a cell displaying a labeling pattern typical of mid S-phase and a measured DNA content of early S phase has completed a cell cycle. The same fraction of γ-H2AX positive cells had completed a cell cycle as the negative or 8H-transfected cell population (Fig. 5D).

To be sure that the lack of cell death and cell cycle arrest in the 32Hc-transfected cells was not due to an eventual impaired response of the cancerous or immortalized cell lines used, we analyzed the response to 32Hc in primary fibroblasts. With as much as 80% γ-H2AX positive cells, the 32Hc-treated population continued to grow in the same way as the untreated population and the cell nuclei, over the three days following transfection, did not show more apoptotic figures than the control (data not shown).

### 32Hc activated DDR response inhibits IRIF formation and disorganizes DNA repair signaling

**Figure 6:**
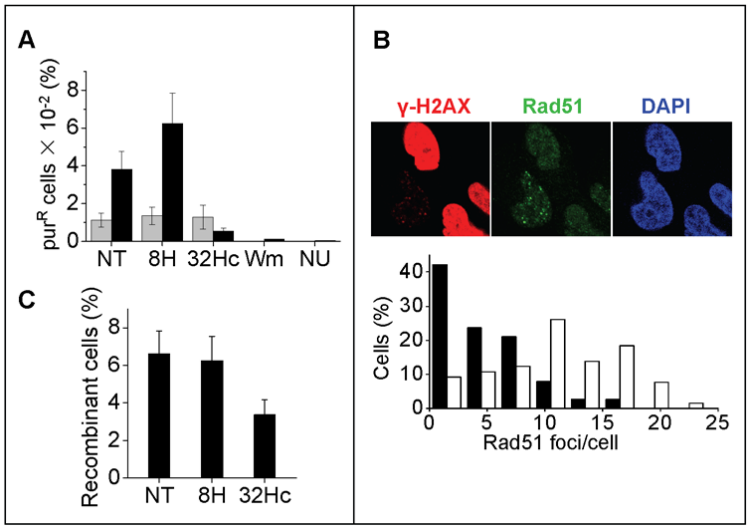
Effect of 32Hc on IRIF formation and DNA repair. (A) Inhibition of radiation-induced plasmid integration. Cells were transfected with 2 µg circular plasmid and 2 µg of 32Hc or 8H. The percentage of pur^R^ cells having integrated the plasmid was calculated after 5 Gy irradiation (black columns) or without irradiation (grey columns) by dividing the number of colony-forming units in the presence of puromycin by the number of colony-forming units in the absence of puromycin for each transfection condition. The values indicated are means of at least three independent experiments. NU, NU7026; Wm, wortmannin. (B) Immunodetection of Rad51 (green) and γ-H2AX (red) in irradiated MRC-5 cells (10 Gy). Cells were fixed 3 h after the end of transfection and IR. DNA was stained with DAPI. Scale bar: 20 µm. Histograms show the quantification (200 cells counted) of the number of foci in γ-H2AX-positive (black) or negative (white) cells. The median values were four foci per γ-H2AX-positive cells and 11 foci per γ-H2AX-negative cells (C) Inhibition of HR. Human RG37 cells containing a tandem repeat of two inactive cassettes coding for EGFP (one of which contains a cleavage site for the meganuclease I-SceI) were transfected with HA-I-SceI expression vector and co-transfected with 8H, 32Hc or not (NT). Recombinant cells express functional EGFP and were measured by flow cytometry. The efficiency of transfection of HA-tagged-I-SceI was followed using an anti-HA antibody and was comparable for all three conditions.

We have recently shown that 32Hc administration prior to irradiation radiosensitizes cells and tumors. Furthermore, IRIF formation of DDR proteins such as 53BP1 and Nbs1 is impaired in 32Hc treated cells indicating an inability of the “positive” cells to undergo efficient damage signaling in response to genotoxic insult [16]. Previous studies were performed with irradiation administered 5 h after beginning of 32Hc treatment. Here we show that this inhibitory effect persists in the γ-H2AX positive cells with the same efficiency in the cells irradiated 24 h after transfection (Supplementary Fig. S4). Consistent with these observations, IRIF formation of the central homologous recombination (HR) protein, Rad51, was inhibited in the γ-H2AX positive cells (Figure 6B). Note also that γ-H2AX did not form foci in the cells with pan-nuclear phosphorylation, indicating that H2AX phosphorylation is saturated. The same observation was made for all other proteins displaying pan-nuclear phosphorylation (not shown).

To analyze whether IRIF formation inhibition affects NHEJ-dependent recombination and HR we analyzed the effect of 32Hc on irradiation-induced plasmid integration [30], [31] and I-SceI induced intrachromosomal recombination [32]. Radiation-induced integration of a plasmid expressing puromycin was inhibited by co-transfection with 32Hc but not with 8H (Fig. 6A) This inhibiting effect was abolished when cells were pretreated with NU7026 or wortmannin. Similarly, recombination between tandem repeats of two inactive genes coding for EGFP was significantly reduced when the I-SceI enzyme coding gene was introduced with 32Hc (Fig. 6C). For this study we used the cell line RG37, in which the pDR-EGFP plasmid is chromosomally integrated as a single copy [33]. After transfection with an expression vector coding for the I-SceI enzyme, recombination induced by the cleavage of the I-SceI site in the SceEGFP cassette recreates a functional EGFP and results in fluorescent cells. With all these analyses we must keep in mind that only approximately 60% of the population is γ-H2AX-positive and that we therefore probably underestimate the effects.

## Discussion

Using short DNA molecules we compared the response triggered by stable DSBs to the DDR response resulting from damage induced by irradiation. We show that 32Hc molecules induce a strong damage signaling that differs from DDR in many ways: (i) the DDR component phosphorylation persists in the cell long after the treatment, (ii) the response displays an “all-or-none” process, (iii) the phosphorylation depends on DNA-PKcs but not ATM and (iv) cell cycle checkpoints and apoptosis do not seem to be activated. In the discussion we will try to comment on each of these differences.

The first aspect we want to describe is the properties of 32Hc molecules as compared to irradiation induced DSBs. 32Hc molecules are blunt-end double stranded DNA protected from degradation by several modifications. Physico-chemical analysis of the molecule revealed that these molecules remain preferentially as duplex DNA, showing high resistance to denaturing conditions and high temperature (T_m_  =  84°C) (unpublished data). Moreover, the phosphorothioate modifications at the free blunt-end prevent extensive degradation by nucleases [17]. In contrast, it has been shown that in irradiated cells, broken DNA is rapidly repaired by NHEJ or extensively processed leading to formation of long 3′ single-stranded DNA regions that will be preferentially repaired by HR. Together with this extensive degradation, small (10-25 bases) oligonucleotides are produced and accumulate in the cell. These degradation products have been shown to trigger ATM-dependent cell cycle checkpoint activation when introduced in *Xenopus laevis* egg extracts [34] and to trigger apoptosis in mammalian cells [35]. Several authors have shown that introducing double-stranded DNA (>200 bp) induced ATM activation [36] whereas short double-stranded DNA molecules had no effect [35]. Since they used unprotected molecules it is very likely that the inducing signal was generated by the degradation products of the long DNA and that short molecules were too rapidly degraded to induce any damage response. The DDR-like activation in response to 32Hc treatment seems to depend exclusively on DNA-PKcs. From this we conclude that the 32Hc molecules persist long enough as blunt double strands in the cells to be recognized by Ku and DNA-PKcs without triggering ATM activity. If a fraction of the 32Hc molecules were degraded, the degradation products would probably be too short to efficiently activate an ATM response.

After 32Hc treatment, we detect a DDR-like activation that lasts for several days. This persistence could be due to the fact that 32Hc molecules are stable and cannot be repaired. Therefore, the continuous damage signaling could counteract the active dephosphorylation by phosphatases described to take place after irradiation [37]-[39]. On the other hand, we cannot exclude that since only a part of the DDR response is turned on and no signal for successful repair would be generated, phosphatases are not activated and the disappearance of the phosphorylated forms is due to natural protein turnover. In agreement with this hypothesis is the observation that the signal persists longer with histone H2AX that is very stable as compared to other proteins such as Rpa32 or ATM. Due to the stability of the signal and the specificity for DNA-PK, 32Hc molecules could become a useful tool to measure DNA-PK activity or inhibition in cells without genotoxic treatment that would compromise cell division and viability.

The “all-or-none” response to 32Hc is more difficult to understand. It seems to correspond to a threshold amount of 32Hc in the cells (Fig. 4). However, fluorescent detection of 32Hc molecules in the cells does not separate biologically active molecules (i.e. molecules able to bind and activate DNA-PK) from inactive molecules degraded or sequestered in endosomes. In our experiments, delivery of 32Hc into the cells was promoted by Superfect or jetPEI polycationic transfection reagents (see Material and Methods). They form small colloidal particles allowing efficient uptake through endocytosis. To test whether the observed “all-or-none” response might be due to a concerted endosomal release in the positive cells or otherwise associated with the transfection agents used, we performed electroporation of MRC-5 cells with naked 32Hc molecules. 32Hc electroporation resulted in the same pan-nuclear distribution of γ-H2AX in a subset of cells as observed with the other transfection agents (Supplementary Fig. S3A). In addition, cells in any stage of the cell cycle were susceptible to triggering a 32Hc-induced response. Thus, the “all-or-none” pattern seems to be a consequence of the partial activation of the DDR by 32Hc that would suppress a negative loop in the regulation of the response rather than a property of the transfection or the state of the recipient cells.

Continuous DNA resection, chromatin remodeling and DNA repair factor assembly at the site of DSBs are necessary to maintain an active checkpoint response in *Saccharomyces cerevisiae* [40]. Since 32Hc molecules are too small to allow IRIF formation, the local concentration and probably crosstalk of the DDR proteins at the signaling locus cannot occur. Actually, the pan-nuclear distribution of the phosphorylated proteins suggests that the spatiotemporal organization of repair proteins is perturbed in 32Hc transfected cells. This is confirmed by the inability of these cells to form IRIF after irradiation. The inhibition of the Rad51 IRIF formation in cells with pan-nuclear H2AX phosphorylation cannot be explained solely by the absence of the γ-H2AX foci signal since H2AX is not essential for the accumulation of Rad51 at DNA damage sites [41]. However, Rad51 binds with higher affinity to the hyper-phosphorylated form of Rpa than native Rpa [42] and foci formation is dependent on the activity of the phosphoinositide-3 family of protein kinases [8] Thus, the ectopic phosphorylation of DNA damage proteins such as Rpa and Nbs1 might perturb the localization of their respective binding partners.

The major endpoints of the DDR response, cell cycle arrest and apoptosis are not reached during the response to 32Hc. Irradiation of 32Hc-treated cells restores both (supplementary Fig, S5, data not shown), indicating that although main proteins as Chk1, Chk2 and p53 are phosphorylated at their main site, part of the signal required for apoptosis and cell cycle arrest is missing after 32Hc treatment and is added by irradiation. Approximately 12 different kinases can phosphorylate p53 and full p53 activity requires at least phosphorylation by both DNA-PK and ATM [43]. It has been suggested that redundant pathways could control risk of erroneous signaling in cells [44]. For example, single-strand DNA (activating ATM/ATR) as well as double strand breaks (activating DNA-PK) could be required for triggering a complete DDR. According to this hypothesis, the signal resulting from 32Hc treatment would not be sufficient to trigger cell cycle arrest and apoptosis because ATM or ATR kinases are not activated.

## Materials and Methods

### Cell culture, Dbait molecules and transfection

Studies of cells in culture were performed using HEp-2 (ATCC number CCL-23), HeLa S3 (ATCC number CCL-2.2), M059K and DNA-PK defective M059J (glioblastomas, [45]), SV40 transformed MRC-5 (ATCC number CCL-171) and AT5BI [46]. DNA-PKcs^-/-^ CHO cells (V3) and V3 cells expressing wild-type (F18) or a kinase-dead domain DNA-PKcs (KA4) [47] were a gift from David J. Chen (Department of Radiation Oncology, University of Texas Southwestern Medical Center, Dallas, TX) and RG37 cells [33] were a gift from B. Lopez (DSV, CEA, Fontenay-aux-roses, France). Human primary foreskin fibroblasts HS68 were purchased from ECACC and used at subconfluence within the first ten passages. Cells were grown at 37°C in monolayer cultures in complete DMEM (Gibco, Cergy Pontoise, France) with 10% FCS and antibiotics (100 µg/ml streptomycin and 100 µg/ml penicillin) under conditions of 100% humidity, 95% air and 5% CO_2_.

Dbait molecules were obtained by automated solid-phase oligonucleotide synthesis from Eurogentec (Seraing, Belgium) as previously described [16]. Sequences are listed in Supplementary Fig. S1.

Transfection of Dbait molecules was performed with Superfect reagent (Qiagen, Courtaboeuf, France) in a ratio of 10 µl Superfect per µg DNA in 1.2 ml medium (in 60 mm diameter plates) for 5 h and then cells were left to recover for 1 h unless otherwise indicated. Transfection with jetPEI (Polyplus-transfection, Illkirch, France) was performed at an N/P ratio of six according to the manufacturer's instructions. For electroporation, 1.2×10^6^ cells were transfected with 2 µg Dbait using the Gene Pulser II (Bio-Rad, Marnes-la-Coquette, France).

### Ku binding and DNA-PK activity assay

^32^P-labeled Dbait molecules were incubated with increasing amounts of HEp-2 nuclear extract (0-320 ng/ml) in 50 mM Tris-acetate pH 7.5, 75 mM potassium acetate, 1 mM EDTA and 1 mM DTT for 10 min at 30°C. Dbait-protein complexes were analyzed on 6% polyacrylamide gels (29∶1). Antibodies to Ku80 (see below) were added prior to gel loading to confirm that the shifted bands contained Ku80 protein.

DNA-PK activity was monitored using the kit SignaTECT DNA-dependent Protein Kinase Assay System (Promega, Madison, WI, USA). The biotinylated peptide substrate, 1.5 µg of nuclear extract (with endogenous DNA removed by DEAE-Sepharose filtration) and 20 nM Dbait molecules were incubated for 5 min at 30°C with (γ-^32^P)ATP according to the manufacturer's instructions. The biotinylated substrate was captured on a streptavidin membrane, washed and counted in a scintillation counter. Percentage of phosphorylation was calculated by dividing the bound radioactivity by the total count of (γ-^32^P)ATP per sample. The DNA-PK inhibitors, NU7026 and wortmannin were purchased from Sigma-Aldrich (St. Louis, MO, USA).

### Recombination assays

NHEJ dependent radiation-induced plasmid integration was measured using a 5,472 bp plasmid derived from pCIEGFP carrying the *puro* gene under control of the SV40 promoter and the gene encoding enhanced green fluorescent protein (EGFP) under control of the CMV1 promoter. Irradiation was performed using a ^137^Cs unit (1.5 Gy/min).

Homologous recombination was monitored using RG37 cells [33] that were co-transfected with 0.5 mg of HA-tagged I-SceI expression vector [48] and 0 or 2 µg of 32Hc or 8H. Recombinant cells were measured by flow cytometry, 72 h after transfection. The efficiency of transfection of HA-tagged-I-SceI was followed using an anti-HA antibody (Cell Signaling Technology, Danvers, MA, USA).

### Antibodies and immunological techniques

Rabbit polyclonal antibodies against the following targets were used: Rad51 (Calbiochem, San Diego, California, USA), Nbs1, Rpa32-S4/8P (Novus Biologicals, Littleton, CO, USA), 53BP1, H2AX, Chk2-T68P, p53-S15P, Nbs1-S343P (Cell Signaling Technology) DNA-PKcs-S2056P (generous gift of David. J. Chen). Rabbit monoclonal Chk1-S345P was purchased from Cell Signaling Technology. The following mouse monoclonal antibodies were used: γ-H2AX (Upstate Biotechnology, Temecula, CA, USA), ATM-S1981P (Abcam, Cambridge, MA, USA), Ku80 (Lab Vision, Fremont, CA, USA), β-actin clone AC-15 (Sigma-Aldrich). For cell cycle experiments, rabbit anti-γ-H2AX (Chemicon-Millipore, Billerica, MA, USA) was incubated for 90 min followed by a 2% PFA fixation, DNA denaturation with 2N HCl for 20 min at RT and extensive washings, then mouse anti-BrdU (Caltag, Bangkok, Thailand) was incubated for 45 min. Nuclei were counterstained with Hoechst 33342 and coverslips were mounted in Vectashield (Vector, Burlingame, CA, USA). Other immunostaining steps were identical to those described in the following section.

Cells grown on coverslips (Menzel, Braunschweig, Germany) were fixed for 15 min in 4% formaldehyde, permeabilized in 0.2% Triton X-100 for 5 min, blocked with 2% BSA and incubated with primary antibody for 1 h at RT or overnight at 4°C. All secondary antibodies conjugated with Alexa-555, Alexa-633, Alexa-488 (Molecular Probes, Eugene, OR, USA), Texas Red (Rockland, Gilbertsville, PA, USA), or Cy3 (Jackson ImmunoResearch, West Grove, PA, USA) were used at a dilution of 1/200 for 30 min at RT and DNA was stained with DAPI.

For immunoblotting, cells were boiled in SDS sample buffer (50 mM Tris-HCl, pH 6.8; 1% β-mercaptoethanol; 2% SDS; 0.1% bromophenol blue; 10% glycerol). Proteins were separated by electrophoresis in 8% or 12% acrylamide/bisacrylamide (35.5/1) gels, transferred to nitrocellulose membranes, blocked with 5% nonfat milk for 1 h and hybridized overnight at 4°C with primary antibody. Blots were then incubated with horseradish peroxidase-conjugated goat anti-rabbit IgG secondary antibodies (P0448, Dako, Glostrup, Denmark) and protein–antibody complexes were revealed on hyperfilm (GE Healthcare, Munich, Germany).

For immunofluorescence detection by flow cytometry, the cells were fixed in 2% para-formaldehyde for 10 min prior to immunodetection. Cells were analyzed by a FACScalibur flow cytometer (BD Biosciences, Franklin Lakes, NJ, USA) and data was analyzed using BD CellQuest Pro (BD Biosciences) and the free WinMDI 2.8 (Scripps Research Institute, La Jolla, CA, USA) software.

### BrdU labeling and microscopy

For BrdU pulse-labeling, 40 µM BrdU (Sigma-Aldrich) was added to the culture medium for 30 minutes and then washed off. Quantitative measurements of DNA content and cell cycle rate were performed *in situ* as previously described [49], [50].

Microscopy analyses were performed at RT using the Leica SP5 confocal system, attached to a DMI6000 stand using a 63×/1.4 objective. Images were processed using the freely available software ImageJ (http://rsb.info.nih.gov.gate1.inist.fr/ij/) complemented with the LOCI bioformat plug-in (http://www.loci.wisc.edu/ome/formats.html) to open images generated by the Leica SP5 confocal system. For microscopical analysis of BrdU stained cells an inverted Leica microscope DMIRE2, equipped for epi-illumination with a 100w mercury arc lamp and 40× oil objective PL Fluotar (NA 0.5-1.0) or 100× oil objective PL APO (NA1.4) was used. Images were captured with a Rooper Coolsnap CCD camera under Metamorph software.

## Supporting Information

Figure S1 List of Dbait molecules.
(0.69 MB TIF)

Figure S2 Effect of inhibitors on 32Hc treatment and necessity of DNA-PK for cell sensitization to irradiation.
(0.76 MB TIF)

Figure S3 TUNEL assay and different transfection protocols.
(1.42 MB TIF)

Figure S4 Persistent inhibition of IRIF formation in γ-H2AX positive cells at 24 h after transfection.
(1.84 MB TIF)

Figure S5 Cell cycle arrest of Dbait treated cells after IR.
(0.60 MB TIF)
