# Supplementary figures and images for: Hyperactivation of DNA-PK by Double-Strand Break Mimicking Molecules Disorganizes DNA Damage Response
Source: PLoS One. 2009 Jul 21;4(7):e6298. doi: 10.1371/journal.pone.0006298 (PMC2709433; doi:10.1371/journal.pone.0006298)

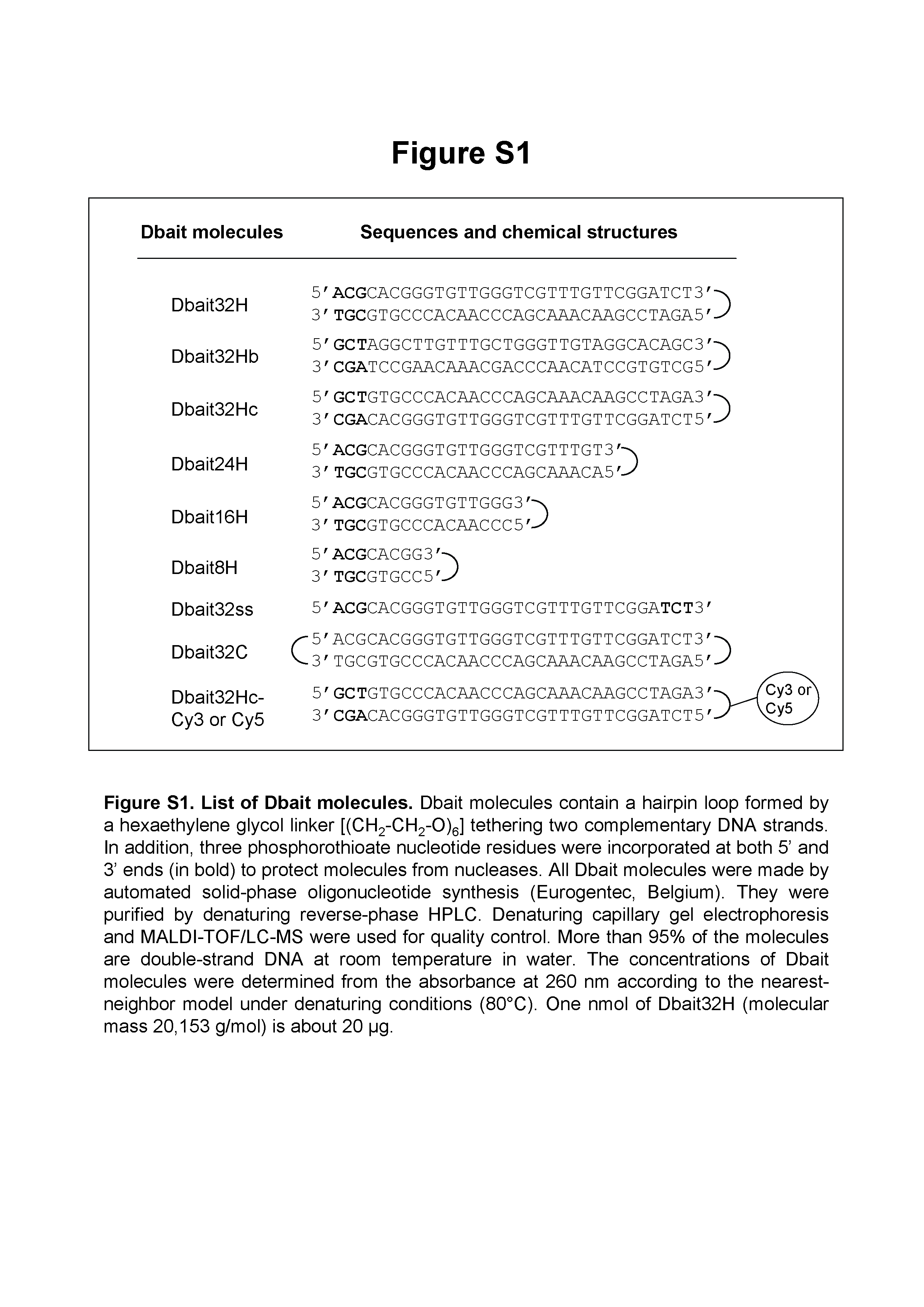

Supplement: Figure S1 — List of Dbait molecules. (0.69 MB TIF) [file pone.0006298.s001.tif]

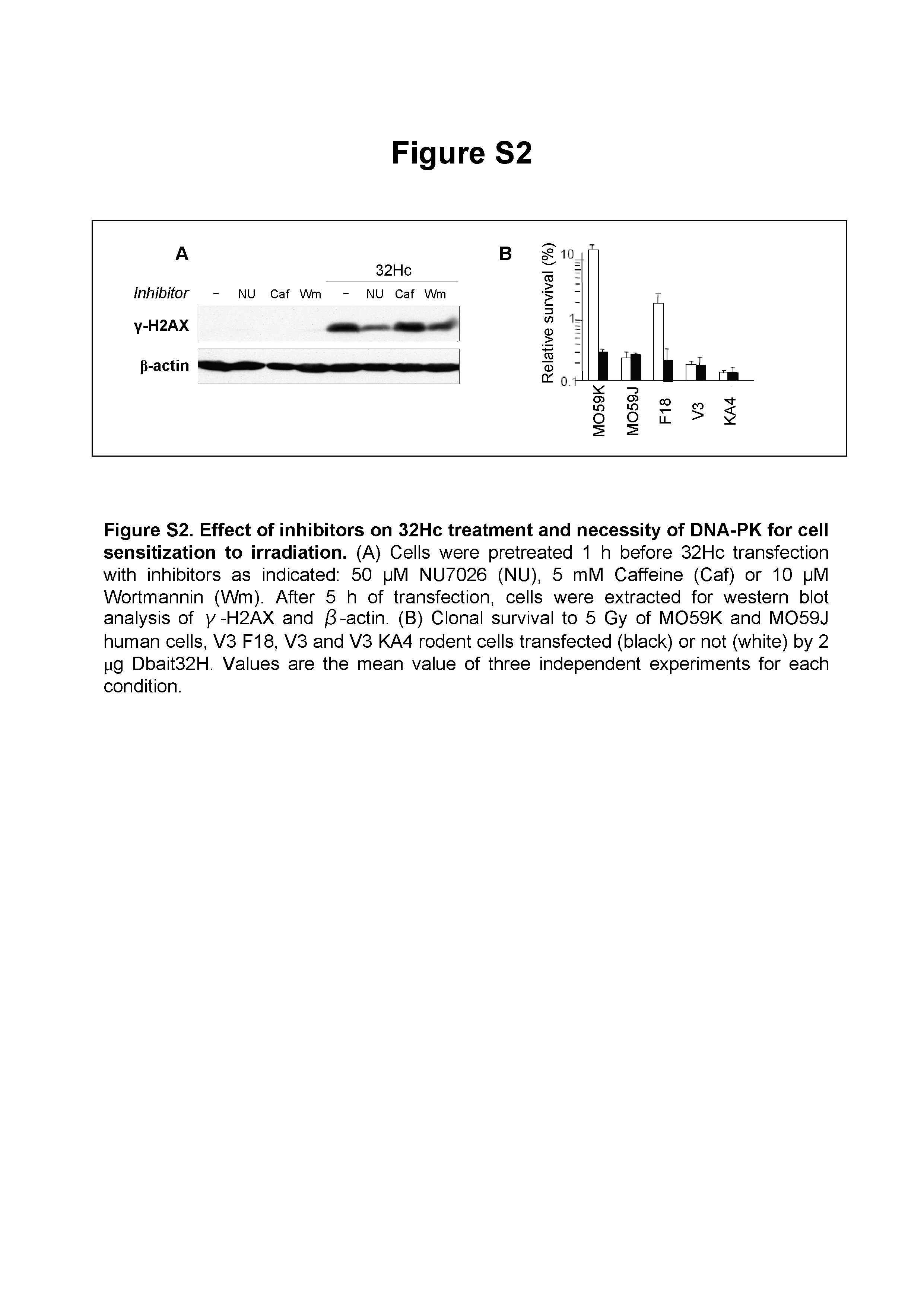

Supplement: Figure S2 — Effect of inhibitors on 32Hc treatment and necessity of DNA-PK for cell sensitization to irradiation. (0.76 MB TIF) [file pone.0006298.s002.tif]

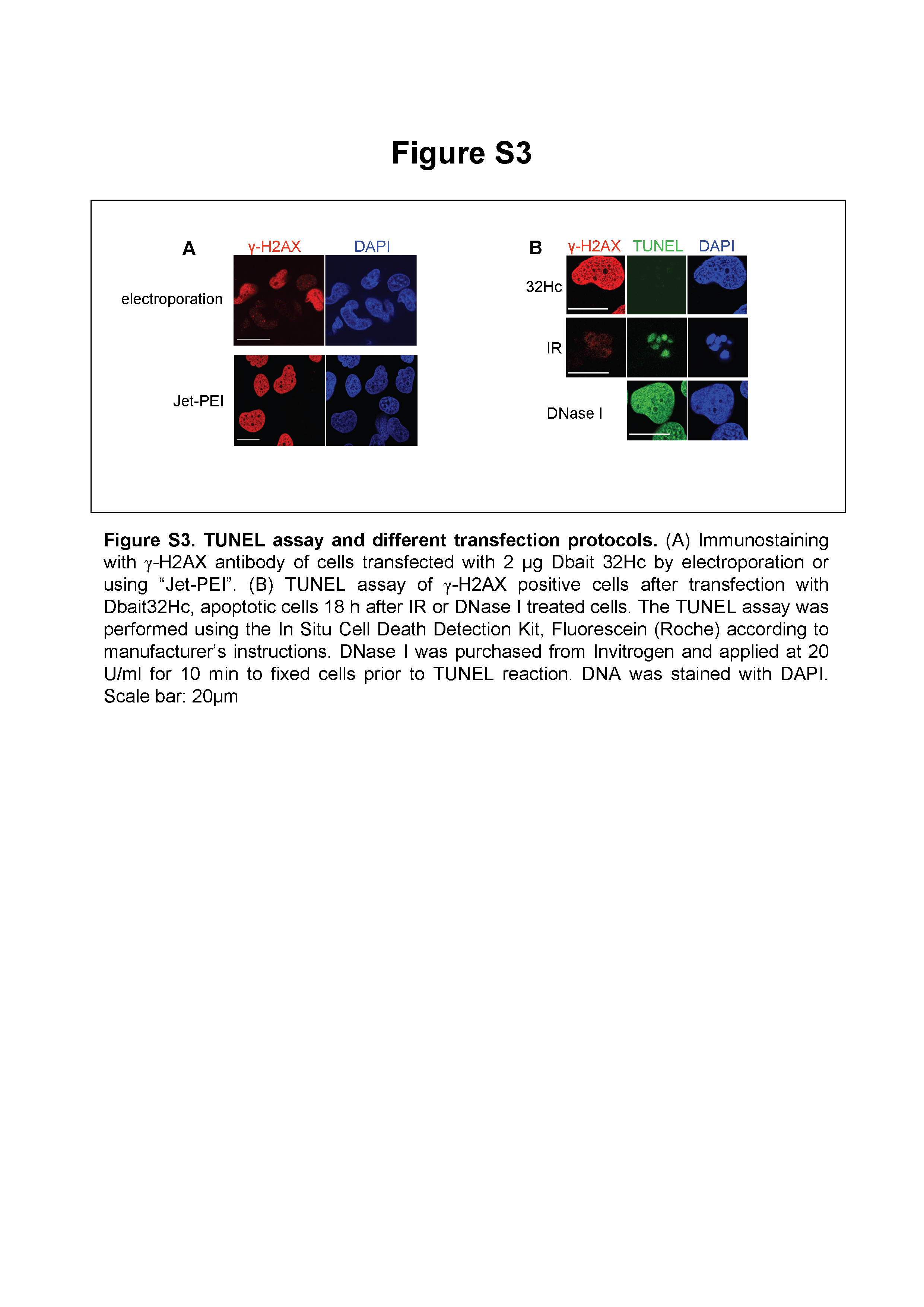

Supplement: Figure S3 — TUNEL assay and different transfection protocols. (1.42 MB TIF) [file pone.0006298.s003.tif]

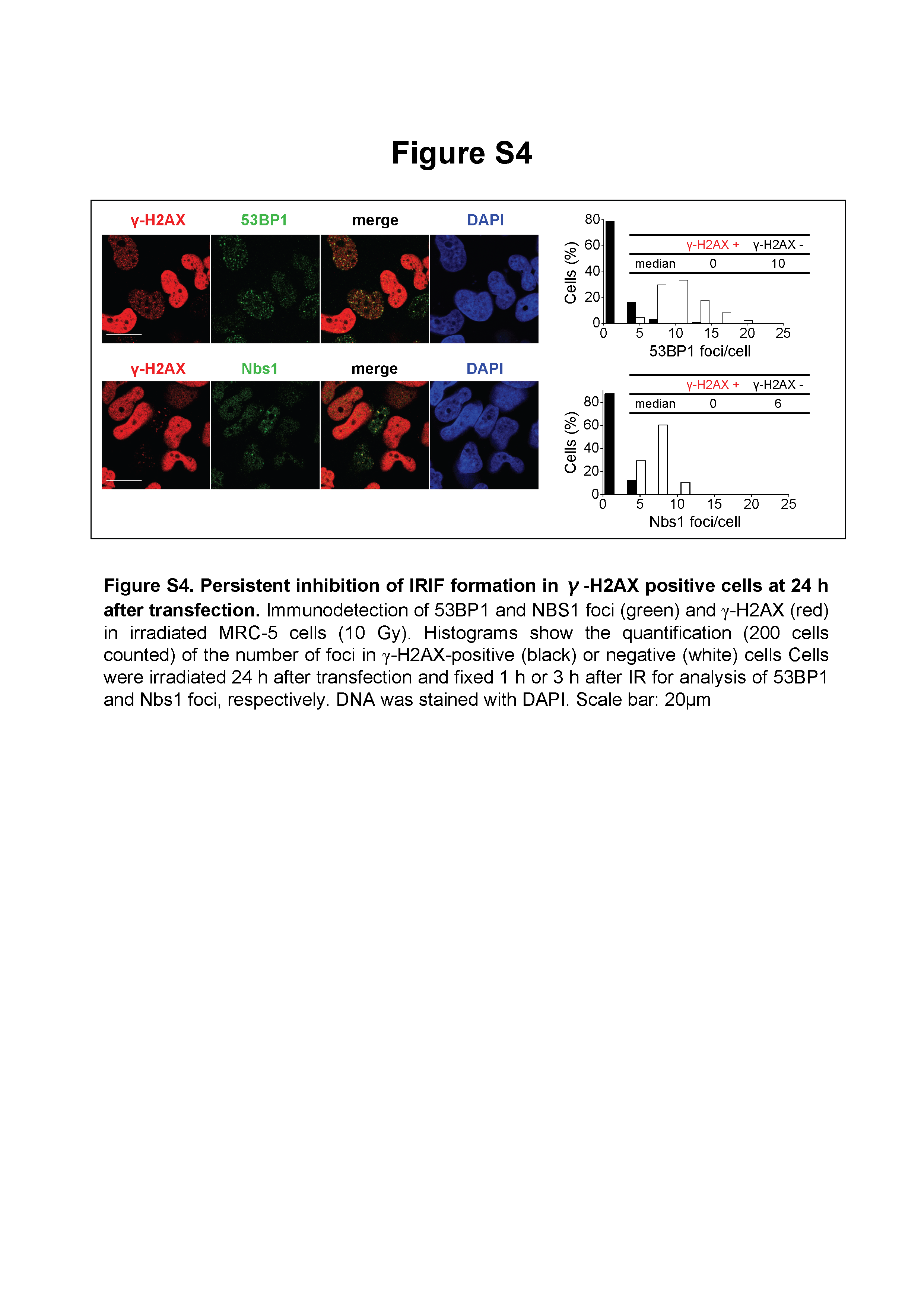

Supplement: Figure S4 — Persistent inhibition of IRIF formation in γ-H2AX positive cells at 24 h after transfection. (1.84 MB TIF) [file pone.0006298.s004.tif]

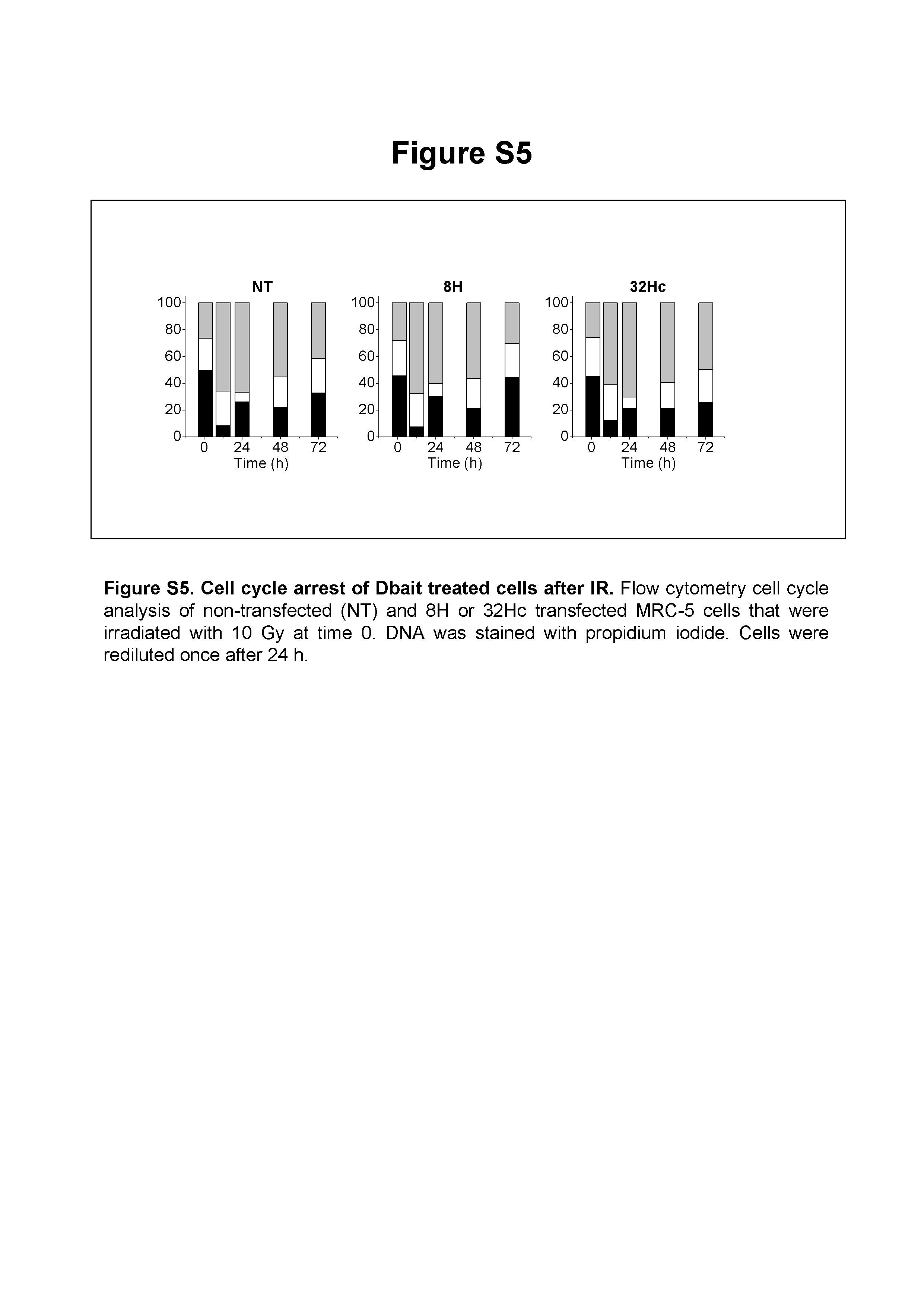

Supplement: Figure S5 — Cell cycle arrest of Dbait treated cells after IR. (0.60 MB TIF) [file pone.0006298.s005.tif]
